# Response of striosomal opioid signaling to dopamine depletion in 6-hydroxydopamine-lesioned rat model of Parkinson's disease: a potential compensatory role

**DOI:** 10.3389/fncel.2013.00074

**Published:** 2013-05-17

**Authors:** Hidetaka Koizumi, Ryoma Morigaki, Shinya Okita, Shinji Nagahiro, Ryuji Kaji, Masanori Nakagawa, Satoshi Goto

**Affiliations:** ^1^Department of Motor Neuroscience and Neurotherapeutics, Graduate School of Medical Sciences, Institute of Health Biosciences, University of TokushimaTokushima, Japan; ^2^Department of Clinical Neuroscience, Graduate School of Medical Sciences, Institute of Health Biosciences, University of TokushimaTokushima, Japan; ^3^Department of Neurology, Graduate School of Medical Science, Kyoto Prefectural University of MedicineKyoto, Japan; ^4^Department of Neurosurgery, Graduate School of Medical Sciences, Institute of Health Biosciences, University of TokushimaTokushima, Japan; ^5^North Medical Center, Graduate School of Medical Science, Kyoto Prefectural University of MedicineKyoto, Japan

**Keywords:** opioid receptors, striosomes, dopamine, Parkinson's disease, striatum

## Abstract

The opioid peptide receptors consist of three major subclasses, namely, μ, δ, and κ (MOR, DOR, and KOR, respectively). They are involved in the regulation of striatal dopamine functions, and increased opioid transmissions are thought to play a compensatory role in altered functions of the basal ganglia in Parkinson's disease (PD). In this study, we used an immunohistochemistry with tyramide signal amplification (TSA) protocols to determine the distributional patterns of opioid receptors in the striosome-matrix systems of the rat striatum. As a most striking feature of striatal opioid anatomy, MORs are highly enriched in the striosomes and subcallosal streak. We also found that DORs are localized in a mosaic pattern in the dorsal striatum (caudate-putamen), with heightened labeling for DOR in the striosomes relative to the matrix compartment. In the 6-hydroxydopamine-lesioned rat model of PD, lesions of the nigrostriatal pathways caused a significant reduction of striatal labeling for both the MOR and DOR in the striosomes, but not in the matrix compartment. Our results suggest that the activities of the striosome and matrix compartments are differentially regulated by the opioid signals involving the MORs and DORs, and that the striosomes may be more responsive to opioid peptides (e.g., enkephalin) than the matrix compartment. Based on a model in which the striosome compartment regulates the striatal activity, we propose a potent compensatory role of striosomal opioid signaling under the conditions of the striatal dopamine depletion that occurs in PD.

## Introduction

The mammalian striatum is not a homogeneous structure but comprises a mosaic organization composed of two functional subdivisions referred to as the striosomes and the matrix compartment (Graybiel, 1990). Neurons in the striosomes and matrix differ in their content of neurotransmitters and receptors, and in their afferent and efferent fiber connections, suggesting interdependent striatal functions of the respective compartments (Graybiel, 1990; Gerfen, 1992). Striosome compartment has been implicated with motor and behavioral brain functions (for review see Graybiel, 2008), and their disorders (Graybiel, 1990; Goto et al., 2010; Crittenden and Graybiel, 2011). Of particular interest, striosomal opioid signaling has emerged as a potent regulator of the striatal activities (Miura et al., 2007, 2008) whereas its functional significance in the pathophysiology of movement disorders remains to be elucidated.

The opioid peptide receptors consist of three major subclasses, namely, μ, δ, and κ, which are encoded by the *OPRM1*, *OPRD1*, and *OPRK1* genes, respectively (Waldhoer et al., 2004; Samadi et al., 2006). Both the μ- and δ-opioid receptors (MORs and DORs, respectively) have an affinity for neuropeptide enkephalin, whereas the κ-opioid receptors (KORs) do for dynorphin (Waldhoer et al., 2004; Samadi et al., 2006). Each type of opioid receptor is differentially localized in the central nervous system and they are implicated in a broad range of brain functions (for review see Mansour et al., 1995). Among the brain regions, the striatum has the highest levels of endogenous opioid peptides and receptors (Mansour et al., 1995; Samadi et al., 2006). All subtypes of opioid receptors are involved in regulating dopamine functions in the brain, and opioid transmissions in the basal ganglia have been implicated in movement disorders such as Parkinson's disease (PD) (Bezard et al., 2003; Samadi et al., 2006). An enhancement of opioid transmission is thought to play a compensatory role in altered functions of the basal ganglia under the conditions of striatal dopamine depletion in PD (Bezard et al., 2003; Samadi et al., 2006).

Using immunohistochemistry with tyramide signal amplification (TSA) protocols, we here determined the distributional patterns of opioid receptors in the striosome-matrix systems of adult rats. We also found that in the 6-hydroxydopamine (6-OHDA)-lesioned rat model of PD, lesions of the nigrostriatal pathways caused compensatory down-regulation of both the MORs and DORs in the striosomes, but not in the matrix compartment. Our results suggest that the striosomes may be more responsive to opioid peptides (e.g., enkephalin) than the matrix compartment. Based on a model in which the striosomes regulates the striatal activity, we also propose that the striosome compartment could be an important anatomical substrate in considering the compensatory roles of enhanced opioid transmissions in PD.

## Materials and methods

### Animals and surgery

All procedures involving experimental animals were approved by the Ethical Review Committee of the University of Tokushima. Male Sprague-Dawley rats (9–10-week-old; Nihon SLC Co., Shizuoka, Japan) were used. Unilateral lesions of the nigrostriatal dopamine pathways were introduced in the rats (*n* = 5) anesthetized with pentobarbital (Sigma, St. Louis, MO), by injecting 6-OHDA (10 μg in saline containing 0.1% ascorbic acid) into the medial forebrain bundle under stereotactic guidance (AP = −4.0 mm, ML = −1.3 mm, DV = −8.4 mm), as previously reported (Crittenden et al., 2009).

### Western blots

Striatal tissues from deeply anesthetized rats were homogenized in 50 mM Tris-HCl buffer, pH 7.5, containing 0.5 M NaCl, 0.5% Triton X-100, 10 mM EDTA, 4 mM EGTA, 1 mM Na_3_VO_4_, 30 mM Na_4_P_2_O_7_, 50 mM NaF, 0.1 mM leupeptin, 0.075 mM pepstatin A, 0.05 mg/ml trypsin inhibitor, 1 mM phenylmethanesulfonyl fluoride, 100 nM calyculin A, and 1 mM dithiothreitol. After centrifugation at 21,500 × *g* for 10 min, the protein lysates were resuspended in 100 mM NaH_2_PO_4_, pH 6.0, 1 mM EDTA, 1% 2-mercaptoethanol, 0.1% sodium dodecyl sulfate, to 1 μg/μl final protein concentration and was heated at 100°C for 3 min. To deglycosylate the opioid receptors, they were then incubated with Endo-β-*N*-acetyl-glucosaminidase F1 (Sigma-Aldrich, St. Louis, MO) at a final concentration of 100 milliunits/ml at 37°C for 16 h. The deglycosylated protein samples were subjected to the trans-immunoblots, according to the method that we previously reported (Yamamura et al., 2013). Specific antibodies against MOR (1:10,000; Millipore, St. Louis, MO), DOR (1:1000; Abcam, Cambridge, UK), and KOR (1:1000; Abcam) were used. The bound antibodies were detected by chemiluminescence staining (ECL plus kit, GE Healthcare, Buckingham, UK). Staining images were captured using a lumino-imaging analyzer LAS-4000 (Fuji, Tokyo, Japan).

### Tissue preparation and immunohistochemistry

Rats with unilateral lesions of the nigrostriatal dopamine pathways (*n* = 5) and normal control rats (*n* = 5) were used. They were injected intraperitoneally with a lethal dose of pentobarbital (Sigma), and were then transcardially perfused with 0.01 M phosphate-buffered saline (PBS) at pH 7.4, followed by cold 4% paraformaldehyde in 0.1 M phosphate buffer (PB) at pH 7.4. The brains were removed, post-fixed overnight in the same fixative at 4°C, and stored in a 10-30% sucrose gradient in 0.1 M PB at 4°C for cryoprotection. Sections were cut on a cryostat at 20 μm-thickness, and stored in PBS containing 0.05% NaN_3_ until use. By using the TSA method, immunostaining was performed on free-floating sections (Okita et al., 2012). Rabbit polyclonal antibodies against MOR (1:100,000; Millipore), DOR (1:10,000; Abcam), KOR (1:10,000; Abcam), tyrosine hydroxylase (TH; 1:100,000) (Sato et al., 2008), and Met-enkephalin (1:50,000; Millipore) were used as primary antibodies. The bound primary antibodies were detected by the Histofine Simple Stain Kit (Nichirei, Tokyo, Japan) and the TSA-system with Cyanine3 or Fluorescein (Perkin Elmer, Shelton, CT). For double labeling, the striatal sections were first stained for MOR using the TSA-system with Fluorescein. To remove the bound antibodies, the stained sections were then incubated in 0.1 M glycine-HCl, pH 2.2, for 30 min. After rinsing with PBS for 1 h, they were labeled for DOR or KOR using the TSA-system with Cyanine3.

### Digital imaging and densitometric analyses

Digital microscopy images were captured using an Olympus BX51 microscope (Olympus, Tokyo, Japan), imported into Adobe Photoshop CS4, and processed digitally for adjustments of contrast, brightness, and color balance. To estimate the density of MOR, DOR, and KOR labeling, the immunostaining of the striatal sections with these antibodies was simultaneously carried out in parallel using the same protocols. By means of Meta Morph (Meta Imaging Series 7.0; Molecular Devices, Tokyo, Japan), the optical densities of immunoreactive products were measured as gray levels on non-colored digital images of striosome and matrix areas in the dorsal striatum, as we previously reported (Sato et al., 2008). For each animal (*n* = 5), measurements were made in 5 dorsal striatal fields at the level of +0.5 to −0.2 mm from bregma according to the atlas of Paxinos and Watson (1997). When striosomes were not clearly visible, they were identified by comparison with the sections doubly stained for MORs.

### Statistical analysis

All experimental values were expressed as means ± S.E.M. Statistical significance was evaluated by the Mann–Whitney *U*-test. The significance level was set at *P* < 0.05.

## Results

### Characterization of antibodies against MOR, DOR, and KOR

**Figure 1:**
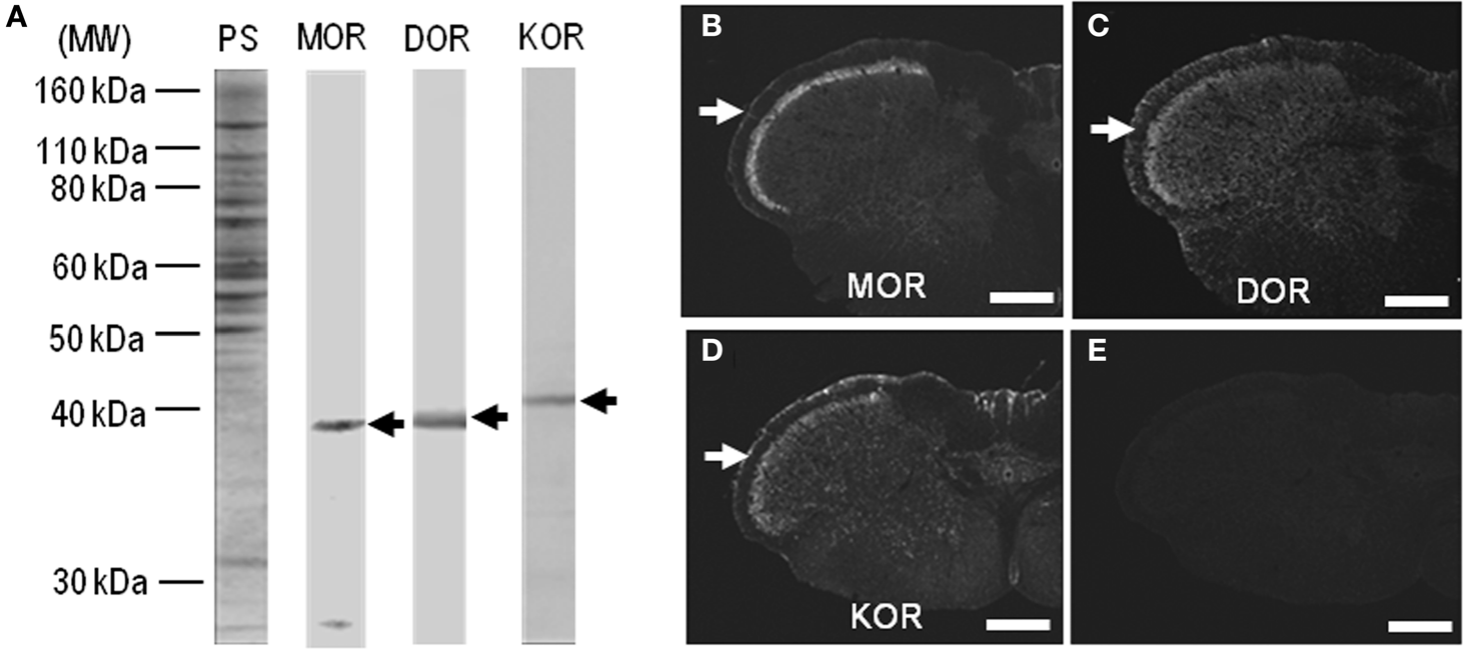
**Characterization of antibodies against opioid receptors. (A)** Rat striatal extracts (10 μg of protein) treated with Endo-β-*N*-acetyl-glucosaminidase F1 (100 milliunits/ml) (see Materials and Methods) were subjected to trans-immunoblots using the antibodies against MOR, DOR, and KOR. Arrows indicate immunoreactive protein bands. MW, molecular weight; PS, protein staining. **(B–D)** Single-label TSA immunostaining of the upper cervical spinal cord by using the antibodies against MOR **(B)**, DOR **(C)**, or KOR **(D)**. Arrows indicate the superficial layers of the dorsal horn of the spinal cord. **(E)** No specific immunoreactivity was found in the spinal cord processed for the TSA protocols in the absence of the primary antibodies. Scale bar = 500 μm.

In this study, we used polyclonal antibodies against C-terminal synthetic peptides for MOR, DOR, and KOR (Table 1). On the immunoblots of rat striatal extracts after the deglycosylation treatment using Endo-β-*N*-acetyl-glucosaminidase F1 (Garzon et al., 1995; Chen et al., 2006; Leskelä et al., 2007), main protein bands with an approximate molecular mass of ~40 kDa, corresponding to the predicted size of the native MOR, DOR, and KOR proteins (Mansour et al., 1995; Waldhoer et al., 2004), were identified (Figure 1). With these antibodies, single-label TSA immunohistochemistry showed heightened immunoreactivity for MOR, DOR, and KOR in the superficial layers of the dorsal horn in the spinal cord (Figures 1B–D), as in a previous report of Schulz et al. (1998). No specific immunoreactivity was found in the spinal cord section processed for the TSA protocols when these antibodies were omitted (Figure 1E). MOR, DOR, and KOR-like immunoreactivity is applied toward all the results, whereas the term “like” is omitted for simplicity.

**Table 1:** **Antibodies against opioid receptors used for immunohistochemistry**.

**Antibody to**	**Immunogen**	**Source**	**Dilution**
μ-opioid receptor (MOR)	Synthetic peptide for the C-terminus of rat μ-opioid receptor	Millipore (St. Louis, MO) Rabbit polyclonal antibody No. AB5511	1:100,000
δ-opioid receptor (DOR)	Synthetic peptide for amino acids 358–372 of rat δ-opioid receptor	Abcam (Cambridge, UK) Rabbit polyclonal antibody No. Ab10272	1:10,000
κ-opioid receptor (KOR)	Synthetic peptide for amino acids 366–380 of human κ-opioid receptor	Abcam (Cambridge, UK) Rabbit polyclonal antibody No. Ab10283	1:10,000

### Neostriatal mosaic distribution of MORs and DORs

**Figure 2:**
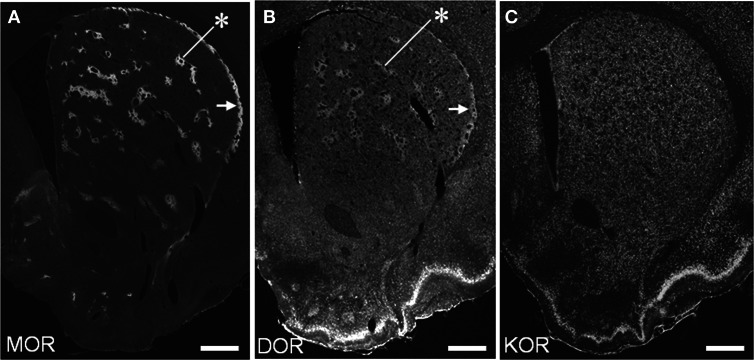
**Distributional patterns of MOR, DOR, and KOR in the striosome-matrix systems of the rat striatum.** Single-label TSA immunostaining of the striatum using the antibody against MOR **(A)**, DOR **(B)** or KOR **(C)**. Asterisks indicate examples of the striosomes, and arrows do the subcallosal streak. Scale bars = 500 μm.

**Figure 3:**
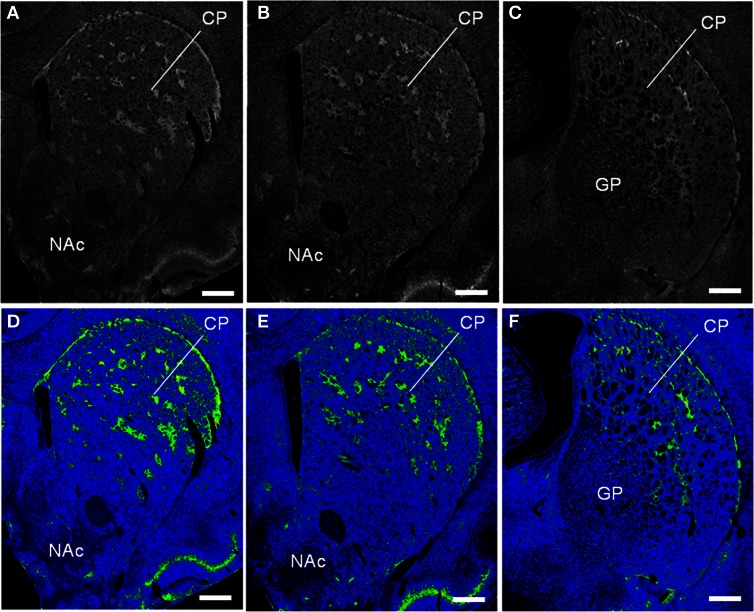
**Striatal patches identified by DOR-immunostaining in the rat striatum.** The frontal sections from anterior to posterior of the striatum labeled for DOR **(A–C)** and their color-converted images **(D–F)**. CP, caudate-putamen; NAc, nucleus accumbens; GP; globus pallidus. Scale bars = 500 μm.

**Figure 4:**
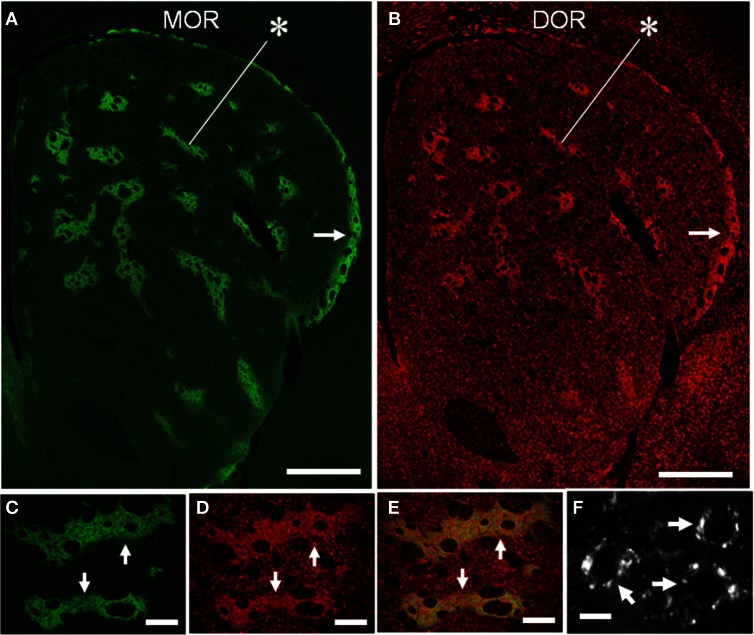
**Compartmental and cellular localization of DORs in the rat striatum. (A,B)** Representative images of striatal sections double-stained for MOR **(A)** and DOR **(B)**. Asterisks indicate a corresponding striosome, and arrows do the subcallosal streak. **(C–E)** Photomicrographs of the striosomes (arrows) double-stained for MOR **(C)** and DOR **(D)**, with merged image **(E)**. DOR labeling is more highly concentrated in the striosomes than in the matrix, although both the compartments contain many cells labeled for DOR. **(F)** A high-power photomicrograph of striosomal cells stained for DORs (arrows). Scale bar: **(A,B)**, 500 μm; **(C–E)**, 100 μm; **(F)**, 10 μm.

We conducted TSA immunohistochemistry to determine the distributional patterns of MORs, DORs, and KORs in the striosome-matrix systems of the rat striatum. Obviously, MOR labeling exhibited a striking mosaic distribution with being highly concentrated in the striosomes and subcallosal streak (Figure 2A) (Pert et al., 1976; Herkenham and Pert, 1981). Similarly, DOR immunoreactivity was also distributed in a “patchy” manner in the striatum (Figure 2B). This mosaic distribution of DORs was found in frontal sections from anterior to posterior of the striatum (Figure 3). When sections were co-labeled for MORs, the DOR patches localized to the striosomes and subcallosal streak identified by MOR immunostaining (Figure 4). However, the DOR patches were obscure in the ventral striatum that includes the nucleus accumbens (Figures 2B and 3). A high-power microscopic image of the striosomal cells stained for the DORs (Figure 4F) showed preferential localization of DOR labeling in their cytoplasm (soma), as consistent with a previous study (Wang and Pickel, 2001). These findings indicate that striosomes and matrix differ in their content of DORs, which results in the enrichment of DORs in the striosome compartment in the dorsal striatum. We also examined the striatal localization pattern of KORs, and found that KOR labeling was virtually homogeneous throughout the dorsal and ventral striatum (Figure 2C).

### Dopamine depletion reduces striosomal MOR and DOR labeling

**Figure 5:**
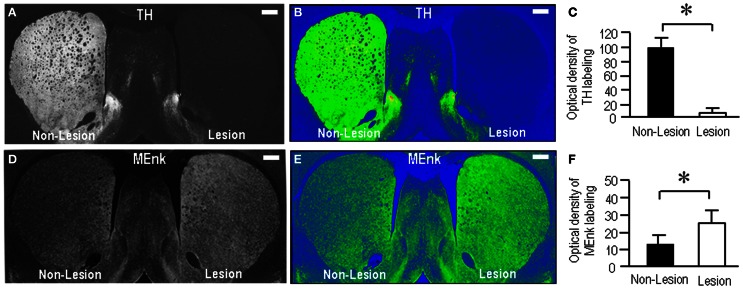
**Rat model with unilateral 6-OHDA lesions of the nigrostriatal pathways. (A,B)** A representative image of the forebrain sections immunostained for TH. Severe loss of TH-immunoreactive afferents is found in the lesioned striatum (Lesion) compared to the non-lesioned striatum (Non-Lesion). Its color-converted image is shown in **(B)**. The intensity of the labeling is shown in a standard pseudocolor scale from blue (lowest labeling) to white (highest labeling) through green, yellow, and red. **(C)** Optical density measurements of striatal TH labeling in the lesioned striatum (Lesion) (*n* = 25) compared to the non-lesioned striatum (Non-Lesion) (*n* = 25). Values are means ± S.E.M. ^*^P < 0.001 (Mann–Whitney *U*-test), Non-Lesion vs. Lesion. **(D,E)** A representative image of the forebrain sections stained for Met-enkephalin (MEnk). An increase in striatal Met-enkephalin labeling is found on the lesioned side (Lesion) compared to the non-lesioned side (Non-Lesion). Its color-converted image is shown in **(E)**. The intensity of the labeling is shown in a standard pseudocolor scale. **(F)** Optical density measurements of striatal Met-enkephalin labeling in the lesioned striatum (Lesion) (*n* = 25) compared to the non-lesioned striatum (Non-Lesion) (*n* = 25). Values are means ± S.E.M. ^*^P < 0.01 (Mann–Whitney *U*-test), Non-Lesion vs. Lesion.

To test the possible interactions between opioid receptors and dopamine depletion in the striatum, we next produced rats (*n* = 5) with unilateral 6-OHDA lesions of the nigrostriatal pathways. Following 3 weeks of recovery, the striatal sections were prepared and processed for immunohistochemical staining. Figures 5A,B illustrates the unilateral lesion of the nigrostriatal dopamine system as demonstrated by the severe loss of labeling for TH, the rate-limiting enzyme in dopamine synthesis, in the lesioned striatum. Optical density measurements revealed a marked (>95%) depletion of TH labeling in the lesioned striatum compared to the non-lesioned striatum (Figure 5C; *P* < 0.001, Mann–Whitney *U*-test). By contrast, Met-enkephalin labeling was increased in the lesioned striatum relative to the non-lesioned striatum (Figures 5D,E), as consistent with a previous report (Dacko and Schneider, 1991). Optical density measurements revealed a significant difference in striatal Met-enkephalin between the lesioned and the non-lesioned sides (Figure 5F; *P* < 0.01, Mann–Whitney *U*-test).

**Figure 6:**
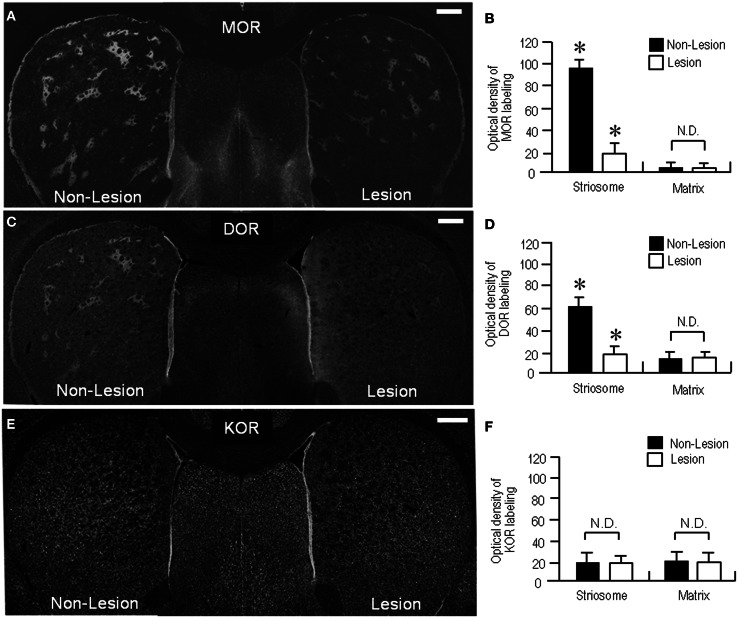
**Loss of striosomal labeling for MOR and DOR in rats with unilateral 6-OHDA lesions of nigrostriatal pathways. (A)** A representative image of the forebrain sections stained for MOR. **(B)** Optical density measurements of MOR labeling in the striosomes and matrix compartment in the dorsolateral portions of the non-lesioned (Non-Lesion) and lesioned (Lesion) striatum. Data are mean ± S.E.M. (bars) values (*n* = 25). ^*^P < 0.005 (Mann–Whitney *U*-test), Non-Lesion vs. Lesion. N.D. indicates no statistically significant difference (*P* > 0.05, Mann–Whitney *U*-test) between Non-Lesion and Lesion. **(C)** A representative image of the forebrain sections stained for DOR. **(D)** Optical density measurements of DOR labeling in the striosomes and matrix compartment in the dorsal striatum of the non-lesioned (Non-Lesion) and lesioned (Lesion) striatum. Data are mean ± S.E.M. (bars) values (*n* = 25). ^*^P < 0.01 (Mann–Whitney *U*-test), Non-Lesion vs. Lesion. N.D. indicates no statistically significant difference (*P* > 0.05, Mann–Whitney *U*–test) between Non-Lesion and Lesion. **(E)** A representative image of the forebrain sections immunostained for KOR. No apparent difference in striatal KOR-labeling is found between the non-lesioned (Non-Lesion) and lesioned (Lesion) sides. **(F)** Optical density measurements of KOR labeling in the striosomes and matrix compartment in the dorsal striatum of the non-lesioned (Non-Lesion) and lesioned (Lesion) striatum. Data are mean ± S.E.M. (bars) values (*n* = 25). N.D. indicates no statistically significant difference (*P* > 0.05, Mann–Whitney *U*-test) between Non-Lesion and Lesion. Scale bars = 500 μm.

**Figure 7:**
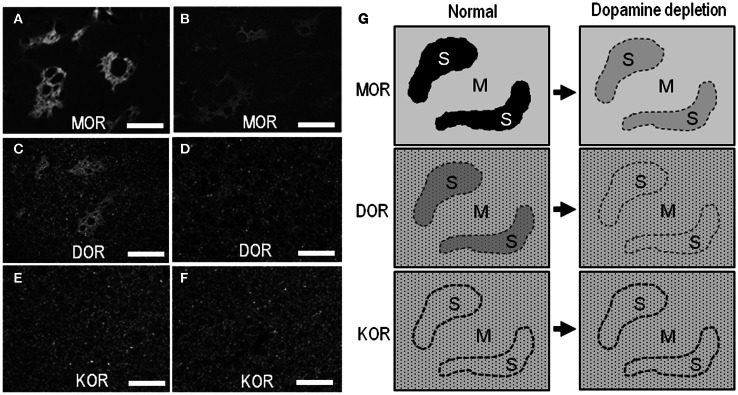
**Light microscopic localization of MOR, DOR, and KOR in the dorsal striatum and its alterations following dopamine depletion. (A–F)** Representative microscopic images of the striatal sections stained for MOR **(A** and **B)**, DOR **(C** and **D)**, and KOR **(E** and **F)** on the non-lesioned **(A,C**, and **E)** and lesioned **(B, D**, and **F)** sides. Scale bar = 200 μm. **(G)** Summary of patterned loss of striatal labeling for MOR, DOR, and KOR under the conditions of dopamine depletion. In normal controls, a heightened labeling for MORs or DORs is found in the striosomes (S). Striatal dopamine depletion causes marked reduction of MOR- and DOR-labeling in the striosomes, but not in the matrix compartment (M). Neuronal cell bodies labeled for DOR or KOR are indicated by small dots.

In agreement with previous studies using the 6-OHDA rat model (Bowen et al., 1982; Johansson et al., 2001), our present study also revealed a reduction of MOR labeling in the dopamine-depleted striatum (Figure 6A). However, it was apparently found in the striosomes and subcallosal streak, but not in the matrix compartment. Similarly, the DOR patches decreased their staining intensities, and thereby, they were obscure in the dorsal striatum on the lesioned side (Figure 6C). These visual impressions were confirmed by quantitative densitometric analyses of immunostained sections. As compared to the non-lesioned striatum, a statistically significant reduction in the levels of staining densities for MOR (Figure 6B; *P* < 0.005, Mann–Whitney *U*-test) and DOR (Figure 6D; *P* < 0.01, Mann–Whitney *U*-test) was noted in the striosomes, but not in the matrix, on the lesioned side. By contrast, striatal KOR labeling did not significantly differ between the non-lesioned and lesioned striatum (Figures 6E,F). Microscopic observations (Figures 7A–F) also showed that dopamine depletion resulted in reduced labeling of DORs, as well as MORs, in the striosomes in the dorsal striatum. Under these conditions in the dorsal striatum, DOR staining was diffusely distributed, as was KOR staining. These findings are summarized in Figure 7G.

## Discussion

In this study, we employed the TSA immunohistochemistry to show the distributional patterns of opioid receptors in the striosome-matrix systems of the rat striatum. Our results showed that like MORs, DORs were highly concentrated in the striosomes, showing a compartmentalized distribution in the dorsal striatum. This notion corroborates with an earliest study using the receptor-binding assay (Bowen et al., 1982), although many subsequent studies have shown a non-patchy and homogeneous distribution of DORs in the striatum, as determined by the receptor-binding (McLean et al., 1986; Mansour et al., 1987; Johansson et al., 2001), *in situ* hybridization (Mansour et al., 1993; Le Moine et al., 1994), and immunohistochemical (Cahill et al., 2001; Wang and Pickel, 2001) assay. On one hand, our data also showed that KOR labeling was virtually homogeneous throughout the striatum. This finding corroborates previous immunohistochemical data (Mansour et al., 1996), but it contradicts the results of a receptor-binding assay (Johansson et al., 2001). We posit that these inconsistent results might depend on the differential assay systems used in individual experiments.

As striatal neurons synthesize and locally express opioid receptors, in particular, MORs and DORs (Mansour et al., 1987, 1994; Guttenberg et al., 1996), our results revealed that striosomal cells exhibited strong DOR-labeling in their cytoplasm. However, previous electron microscopic immunocytochemical studies showed that in striatal patches, DORs are also present in the axon terminals that form symmetric synapses, suggesting that DORs play a role in modulating the presynaptic release of excitatory amino acids such as glutamate (Wang and Pickel, 2001). This is supported by electrophysiological evidence that the DOR agonists inhibit glutamatergic inputs to medium spiny neurons (MSNs) by acting at presynaptic sites in the striatum (Jiang and North, 1992; Blomeley and Bracci, 2011). It is therefore suggested that in striatal patches, DORs are present not only in the striosomal cells and their local projections but also in the corticostriatal afferent nerve terminals, as are MORs (Jiang and North, 1992; Wang and Pickel, 2001; Blomeley and Bracci, 2011). At the cellular level, MORs are present largely in the MSNs bearing dopamine D1 receptors (D1Rs) in the striosomes (Delfs et al., 1994; Guttenberg et al., 1996; Georges et al., 1999; Wang et al., 1999). It remains unclear whether DORs are colocalized with MORs in such a subset of striosomal MSNs. Given that striosomes are enriched in both the MORs and DORs relative to the matrix, our findings indicate a compartmental difference in the regulation of opioid signaling by these opioid receptors. This, in turn, suggests the striosomes may be more responsive to opioid peptides (e.g., enkephalin) than the matrix compartment.

It has so far been disputed that in PD, an enhancement of opioid transmission might play a compensatory role in altered functions of the basal ganglia (Bezard et al., 2003; Samadi et al., 2006). However, the precise mechanism by which the increased opioid signaling modulates the basal ganglia activity is still under debate. One of the most recognized models of functional organization of the basal ganglia indicates a key role for balance in the activity of the two major striatal output pathways, i.e., the direct and indirect pathways (Alexander and Crutcher, 1990). Dopamine depletion is known to cause increased opioid transmission in the striatum (Miura et al., 2008). Strikingly, this is found in MSNs that form the indirect pathway (the indirect-pathway MSNs); the level of expression of enkephalin and *preproenkephalin (PPE)*-A mRNA is increased in the indirect-pathway MSNs (Morissette et al., 1999; Quik et al., 2002; Meissner et al., 2003), whereas that of dynorphin and *PPE-B* mRNA was unaltered or decreased in the direct-pathway MSNs (Cenci et al., 1998; Meissner et al., 2003). The pronounced upregulation of enkephalin in the indirect-pathway MSNs can cause a compensatory down-regulation of both MORs and DORs in their target cells (Höllt, 1986). This agrees with findings that prolonged activation of MORs and DORs by the opioid ligand (i.e., enkephalin) promotes their proteolytic degradation process that contributes to homeostatic regulation of cellular opioid responsiveness (Henry et al., 2011; Hislop et al., 2011). Indeed, we here showed a striking reduction of both the MORs and DORs in the 6-OHDA lesioned striatum that exhibited the increased level of expression of enkephalin, as consistent with previous studies (Bowen et al., 1982; Trovero et al., 1990; Smith et al., 1993; Johansson et al., 2001; Jabourian et al., 2007). However, our immunohistochemical data also revealed that it was significantly found in the striosomes, and suggest the possibility that the striosome compartment might implicate with the compensatory role of increased opioid transmissions in PD.

**Figure 8:**
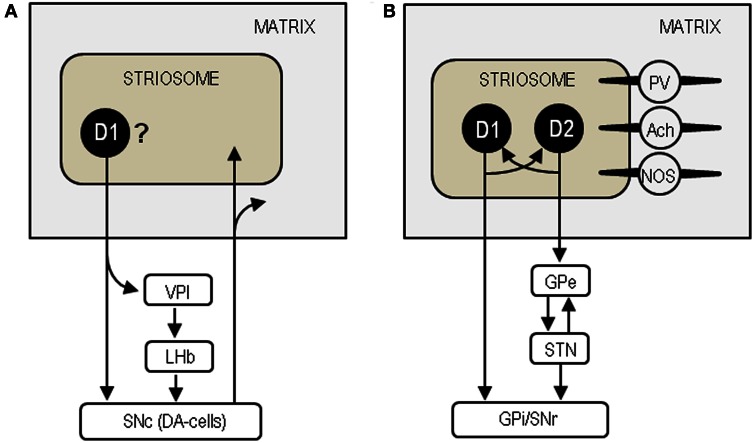
**Neural circuits based on the striosome compartment. (A)** Model of the striosome-SNc pathways. SNc, substantia nigra pars compacta; DA-cells, dopamine-producing cells; VPI, ventral pallidum intermediate; LHb, lateral habenula. **(B)** Model of the intercompartmental communication via striatal interneurons. D1, dopamine D1 receptor; D2, dopamine D2 receptor; GPe, globus pallidus externa; STN, subthalamic nucleus; GPi, globus pallidus internus; SNr, substantia nigra pars reticulata; PV, parvalbumin; Ach, acetylcholine; NOS, nitric oxide synthase.

There is a large body of evidence suggesting that striosomal MSNs may be unique among striatal cells in sending their GABAergic projections directly to the substantia nigra pars compacta (SNc), which contains dopamine-producing cells (DA-cells) that project back to both the striosome and matrix compartments (see Figure 8A) (Gerfen, 1984; Jimenez-Castellanos and Graybiel, 1989; Tokuno et al., 2002; Fujiyama et al., 2011). They also reportedly innervate specifically the regions of the GPi (i.e., the ventral pallidum intermediate) that in turn project to the lateral habenula, which can inhibit the activity of DA-cells in the SNc (Herkenham and Nauta, 1979; Christoph et al., 1986; Ji and Shepard, 2007; Matsumoto and Hikosaka, 2007; Bromberg-Martin et al., 2010). Thus, the striosome compartment could be in a position to exert global control over dopamine signaling in the dorsal striatum (Crittenden and Graybiel, 2011). Given the general inhibitory effect of opioid peptides (Samadi et al., 2006), increased opioid activities due to dopamine depletion in the striosomes may reduce the GABAergic (inhibitory) outputs of striosomal cells. This would effectively disinhibit and activate the residual DA-cells in the SNc to increase dopamine release in the striatum. We thus hypothesize that enhanced opioid transmission in the striosomes could exert a compensatory role via the striosome-SNc pathways in the parkinsonian state.

Striosomes are also thought to communicate with the matrix compartment via striatal interneurons that include cholinergic interneurons, GABAergic fast-spiking interneurons containing parvalbumin, and GABAergic low-threshold spiking interneurons containing nitric oxide synthase, somatostatin and neuropeptide Y (see Figure 8B) (for review see Miura et al., 2008; Crittenden and Graybiel, 2011). Among these interneurons, cholinergic cells are particularly interesting because they can control dopamine release and their overactivity causes motor impairments in PD (Pisani et al., 2007; Aosaki et al., 2010). The direct- and indirect-pathway MSNs in the striosomes are thought to regulate the activities of the matrix MSNs by modulating acetylcholine release from cholinergic cells (Miura et al., 2008). Via their synaptic contacts that transmit substance P, the direct-pathway MSNs could strongly depolarize cholinergic cells to evoke acetylcholine release (Aosaki et al., 2010). On one hand, the indirect-pathway MSNs containing enkephalin could presynaptically suppress the activities of the direct-pathway neurons by activating the MORs and DORs on them (Aosaki and Kawaguchi, 1996). Accordingly, in the parkinsonian state, loss of the D1R-mediated signals could inactivate the direct-pathway MSNs in the striosomes, thereby reducing acetylcholine release from cholinergic cells. Simultaneously, loss of the D2R-mediated signals could disinhibit and activate the indirect-pathway MSNs to facilitate enkephalin release, leading to further inactivation of the direct-pathway MSNs. Moreover, the increased opioid transmission in the striosomes also may directly reduce striatal cholinergic activity because enkephalin can hyperpolarize cholinergic cells by activating their DORs and then stop acetylcholine release (Jiang and North, 1992). Collectively, we suggest that enhanced opioid signaling in the striosome compartment may also exert a compensatory role by reducing the activity of cholinergic cells in PD.

In conclusion, we here showed that in the dorsal striatum of rats, the striosome compartment is enriched in both the MORs and DORs, suggesting that the striosomes might be more responsive to opioid peptides (e.g. enkephalin) than the matrix compartment. This notion is in accordance with the present finding that in the 6-OHDA model of PD, compensatory down-regulation of MORs and DORs occurs in the striosomes. Based on a model in which the striosome compartment regulates the activity of the matrix compartment that plays a major role in the striatal output systems, we hypothesize that striosomal opioid signaling could exert a potent compensatory regulation of the matrix activity via striosome-SNc projections and intercompartmental communication. A further understanding of the cellular and molecular mechanisms involved in striosomal opioid signaling might provide novel strategies to treat PD and associated motor complications such as levodopa-induced dyskinesia.

### Conflict of interest statement

The authors declare that the research was conducted in the absence of any commercial or financial relationships that could be construed as a potential conflict of interest.
